# Investigation of non-motor symptoms in patients with amyotrophic lateral sclerosis

**DOI:** 10.1007/s13760-022-02036-6

**Published:** 2022-08-20

**Authors:** Takehisa Hirayama, Mari Shibukawa, Masaru Yanagihashi, Hitoshi Warita, Naoki Atsuta, Koji Yamanaka, Osamu Kano

**Affiliations:** 1 Department of Neurology, Faculty of Medicine, Toho University, Tokyo, Japan; 2 Department of Neurology, Tohoku University Hospital, Sendai, Japan; 3 Department of Neurology, Aichi Medical University School of Medicine, Nagakute, Japan; 4 Department of Neuroscience and Pathobiology, Research Institute of Environmental Medicine, Nagoya University, Nagoya, Japan

**Keywords:** Amyotrophic lateral sclerosis, Non-motor symptoms, Quality of life, Fatigue, Pain

## Abstract

[Objective] Few studies have comprehensively investigated the non-motor symptoms of amyotrophic lateral sclerosis (ALS). We aimed to investigate this aspect of ALS. [Methods] We held a nationwide webinar, titled “ALS Café,” and distributed self-report questionnaires to ALS patients. In addition to the frequency of non-motor symptoms such as fatigue, pain, sleep disorders, defecation disorders, sialorrhea, and sexual problems, we evaluated the quality of life (QoL), ALS Functional Rating Scale-Revised (ALSFRS-R), and Patient Health Questionnaire-9 (PHQ-9). [Results] The average age of the 33 respondents (19 male, 14 female) was 60.8 ± 11.2; 96.7% of respondents had some non-motor symptoms. The median ALSFRS-R was 32.0, and seven (21.2%) of the respondents had a PHQ-9 score of 10 or higher. Fatigue was the most common non-motor symptom (81.8%), followed by pain (60.6%), defecation disorders (57.6%), sleep disorders (48.5%), sialorrhea (48.5%), and sexual problems (24.2%). Fatigue was more frequent in females (*P* = 0.03). Among the non-motor symptoms, pain was the most common factor affecting QoL, followed by fatigue. More than 90% of ALS patients answered that they had never consulted a physician/counselor about sexual problems. Patients with pain had higher PHQ-9 scores than those without (*P* = 0.01). There was no correlation between the ALSFRS-R score and QoL and PHQ-9. [Conclusions] Most patients with amyotrophic lateral sclerosis had non-motor symptoms, and fatigue and pain were the most common. We showed that many non-motor symptoms affected QoL without correlating with ALSFRS-R score. Attention should be paid to those even if the motor symptoms of ALS are mild.

## Introduction

Amyotrophic lateral sclerosis (ALS) is a degenerative disease that involves central and peripheral motor neurons. Without a ventilator, respiratory and swallowing muscle weakness can lead to death within 3–5 years of onset [1]. Therefore, treatment and symptom assessment are often focused on motor symptoms and survival. However, non-motor symptoms, such as pain, fatigue, and sleep disorders, are observed in patients with ALS, and these symptoms reduce their quality of life (QoL) [2]. Many of these non-motor symptoms are poorly defined with unexplained mechanisms, are underdiagnosed, and remain unreported during clinical consultation [2, 3]. Additionally, ALS patients experiencing a greater frequency of non-motor symptoms have a lower quality of life than those who indicate more severe motor symptoms [4], which have been repeatedly reported as impactful and poorly evaluated in patients with ALS. Non-motor symptoms of ALS have not been comprehensively or consistently evaluated in clinical trials [5]. However, a weakness of that study was its focus on a limited number of non-motor symptoms in ALS, namely neuropsychiatric, cognitive, and behavioral changes, pain, disordered sleep, fatigue, and sialorrhea. However, non-motor symptoms in ALS are diverse, and it is important to investigate the actual state of a wider range of non-motor symptoms, such as gastrointestinal and sexual dysfunction. Therefore, in this study, we comprehensively investigated the non-motor symptoms of patients with ALS, reported their characteristics, and assessed their impact on QoL.

## Methods

This survey research was approved by the Ethics Committee of Toho University Omori Hospital, Tokyo, Japan (reference no. M20020). In June 2021, we held a nationwide webinar titled “ALS Café” for patients with ALS, along with their families and caregivers in the ALS multidisciplinary clinic of Toho University Omori Medical Center, wherein self-report questionnaires were distributed to the participants. There was a total of 328 connections with communication equipment, including patients with ALS, their families, doctors, nurses, and other healthcare professionals. At the end of the program, we conducted a survey on matters related to non-motor symptoms using Google Forms (Google, Menlo Park, CA). Specifically, we asked patients regarding their non-motor symptoms, such as pain, fatigue, sleep disorders, defecation disorders, excessive salivation, and sexual problems, and subsequently examined the relationship between these symptoms and depression. Depression was evaluated using the Patient Health Questionnaire-9 (PHQ-9), with a cut-off score of 10 points (higher values indicating depression) [6, 7]. Furthermore, we asked patients regarding the severity of these symptoms using the ALS Functional Rating Scale-Revised (ALSFRS-R), wherein each item in the questionnaire was explained prior and patients were evaluated based on their points [8]. The questions on QoL had four options: a significant decrease in QoL, a decrease in QoL, a mild decrease in QoL, and no decrease in QoL. For assessment, "significant decrease in QoL" and "decrease in QoL" were combined as decrease in QoL. We then investigated the proportion of patients with decreased QoL due to their symptoms. For clarification, we described in the questionnaire that sexual problems included not only sexual function disturbances, such as erectile dysfunction and amenorrhea, but also troubles with sexual activity and sexual satisfaction. Furthermore, for each non-motor symptom, we compared gender differences, as well as differences in the ALSFRS-R and PHQ-9 scores depending symptomology. For statistical analysis, we performed Fisher’s exact test for categorical data and Mann–Whitney U test for continuous data. Spearman’s rank correlation test was used to evaluate correlations between the ALSFRS-R and PHQ-9 scores. Statistical significance was set at *P* < 0.05, and all statistical analyses were performed using the EZR (Saitama Medical Center, Jichi Medical University, Saitama, Japan) software, which is a graphical user interface for R (The R Foundation for Statistical Computing, Vienna, Austria) [9].

## Results

**Fig. 1:**
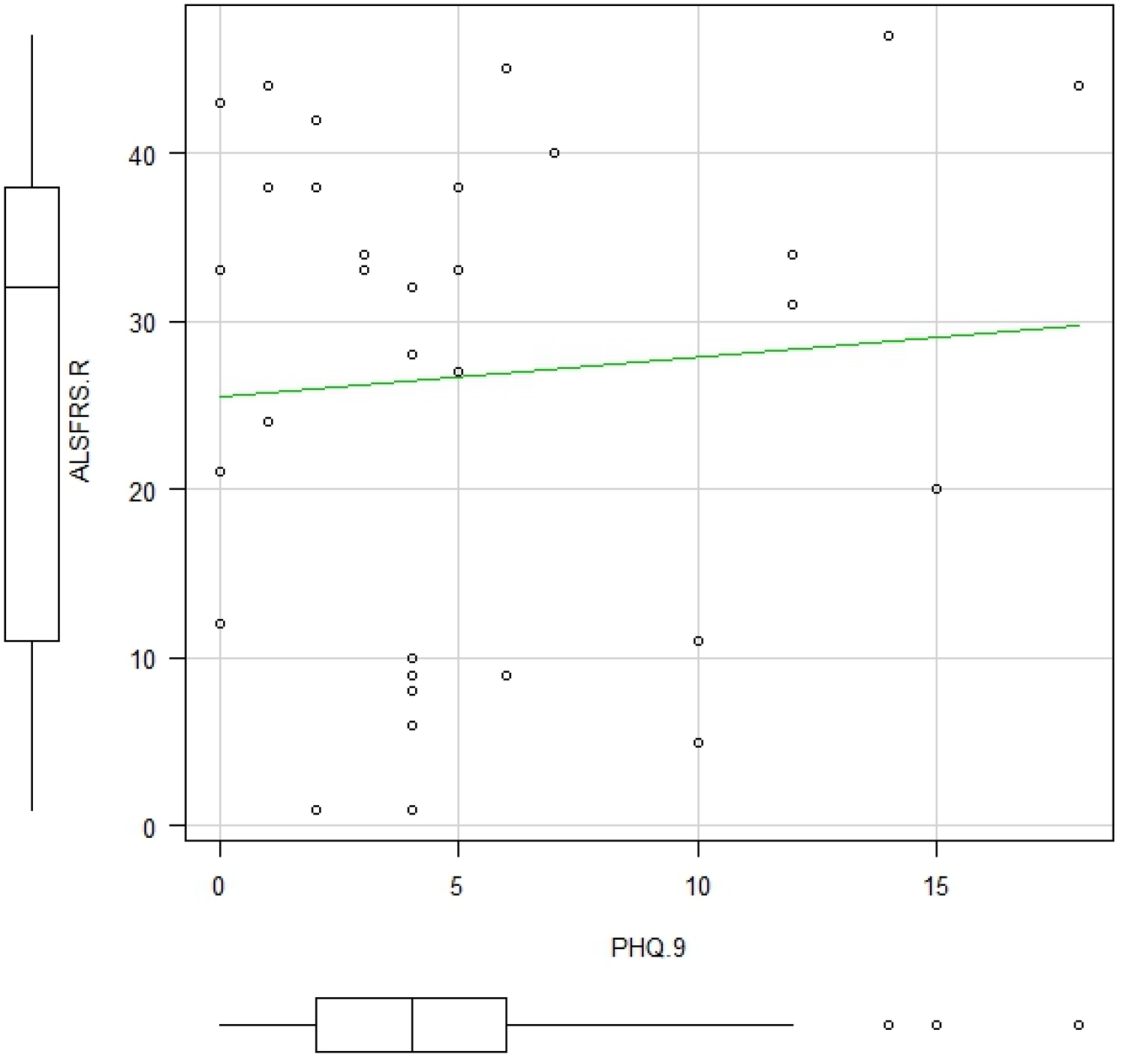
Relationship between the ALSFRS-R and PHQ-9 scores. Spearman’s rank correlation test was used to evaluate correlations between the ALSFRS-R and PHQ-9 scores. The correlation coefficient was calculated as -0.00876 (*p* value = 0.961). No correlation was found between the ALSFRS-R and PHQ-9 scores

**Fig. 2:**
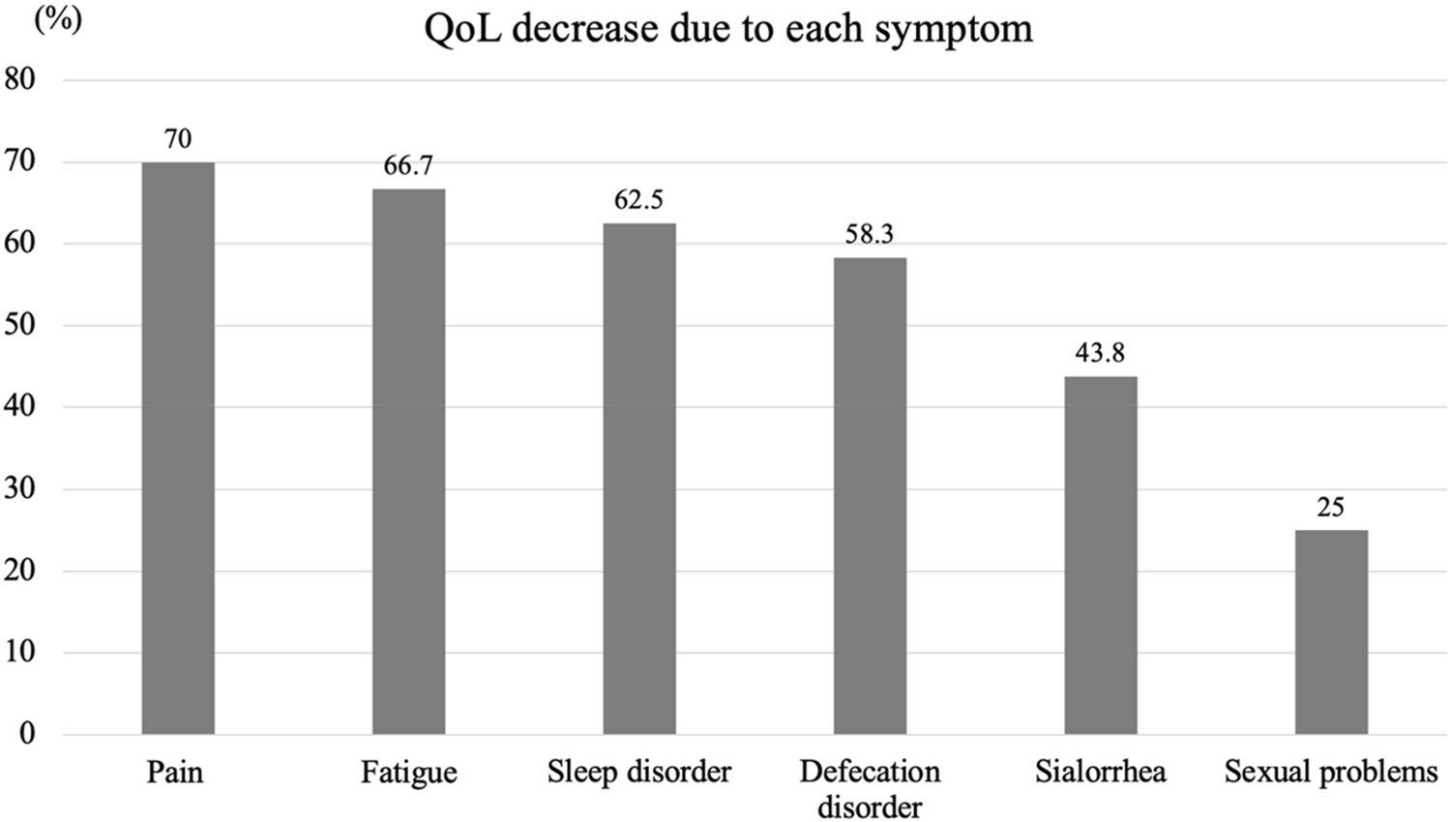
Percentage of patients with each non-motor symptom who reported a decrease in quality of life (QoL). Pain was the most common non-motor symptom, which was followed by fatigue. Approximately 70% of patients with non-motor symptoms answered that their QoL was decreased, including approximately 60% of patients with defecation and sleep disorders

We received responses from 33 patients with ALS (19 males, 14 females), with an average age of 60.8 ± 11.2 years. The respondents had the following comorbidities: ischemic heart disease (*n* = 1), diabetes (*n* = 1), diabetes and bronchial asthma (*n* = 1), hypertension (*n* = 2), dyslipidemia (*n* = 1), and hypertension and dyslipidemia (*n* = 1). Respondent characteristics and their responses to the questions are presented in Table 1. All but one respondent had some non-motor symptoms, and 93.9% of ALS patients had multiple non-motor symptoms. Among them, 75.6% of patients with non-motor symptoms reported a decrease in QoL due to their respective symptoms. The median ALSFRS-R score was 32.0, and seven (21.2%) of the respondents had a PHQ-9 score of 10 or higher. Four patients (two males and two females) who underwent tracheostomy and invasive ventilation (TIV) were included. As shown in Table 2, fatigue was the most common non-motor symptom (81.8%), followed by pain (60.6%), defecation disorders (57.6%), sleep disorders (48.5%), sialorrhea (48.5%), and sexual problems (24.2%). There were two patients with ALS who had pressure ulcers. Their ALSFRS-R scores were 5 and 11 points and they had no underlying disease other than ALS. On comparison, there was a difference in patients with fatigue based on sex (Table 2), wherein more females suffered from fatigue (*P* = 0.03). Patients with pain also had significantly higher PHQ-9 scores than those without pain (*P* = 0.01), and patients with sialorrhea had lower ALSFRS-R scores (*P* = 0.03). We did not find any correlations between ALSFRS-R and PHQ-9 scores (Fig. 1), and there were no differences in the ALSFRS-R scores between the group with a PHQ-9 score of ≥ 10 and the group with a PHQ-9 score of < 10 (Table 3). Among the non-motor symptoms, pain was the most common factor affecting QoL (70.0%), followed by fatigue (66.7%), sleep disorders (62.5%), defecation disorders (58.3%), sialorrhea (43.8%), and sexual problems (25.0%) (Fig. 2). Further details regarding these non-motor symptoms are presented in Table 4. Pain as a non-motor symptom was most often attributed to muscle cramps and compression due to immobility (40.0%), which was followed by hypertonia (35.0%). The most common cause of sleep disorders was unknown (56.3%), which was followed by muscle spasms (31.3%). Regarding defecation disorders, constipation was the most common (68.4%), which was followed by lack of abdominal pressure (57.9%). Although the median ALSFRS-R score was described, the population parameter was small for fecal incontinence, no urge to defecate, and diarrhea. Therefore, these scores were not available. On the other hand, the median ALSFRS-R score for those who answered that they had pressure due to immobility and lack of abdominal pressure was 9.5. Sexual problems were reported by 5 of the 11 patients aged < 60 years (45.5%), 2 of the 3 patients aged < 40 years (66.7%), and 4 of the 22 patients aged ≥ 60 years (18.2%). However, since the population parameter under 50 years of age was very small (1 person), the percentage of those with sexual problems was not available. In addition, more than 90% of patients answered that they had never consulted anywhere about sexual problems. There was no difference in ALSFRS-R score between the group reported decrease in QoL and no decrease in QoL, whereas patients with ALS who answered that they had a decrease in QoL had high PHQ-9 scores.

**Table 1:** Respondent characteristics

ALS patients (*n* = 33)	
Age, years	60.8 ± 11.2
Female,* n* (%)	14 (42.4)
ALSFRS-R, median values	32
Visiting ALS Multidisciplinary clinic,* n* (%)	13 (39.4)
With caregivers,* n* (%)	31 (93.9)
Any non-motor symptoms	32 (96.7)
Multiple non-motor symptoms	31 (93.9)
Decrease in QoL due to one or more non-motor symptoms	25 (75.8)

*ALS* Amyotrophic lateral sclerosis, *ALSFRS-R* Amyotrophic lateral sclerosis Functional Rating Scale-Revised, *QoL* Quality of life, *PHQ-9* Patient Health Questionnaire-9

**Table 2:** Sex difference of non-motor symptoms

	ALS patients (*n* = 33)	Male (*n* = 19)	Female (*n* = 14)	*P* value^a^
Fatigue, (%)	27 (81.8)	13 (68.4)	14 (100)	**0.027**
Pain, (%)	20 (60.6)	9 (47.4)	11 (78.6)	0.087
Defecation disorder, (%)	19 (57.6)	11 (57.9)	8 (57.1)	1.000
Sleep disorder, (%)	16 (48.5)	8 (42.1)	8 (57.1)	0.491
Sialorrhea, (%)	16 (48.5)	9 (47.4)	7 (50)	1.000
Sexual problems, (%)	8 (24.2)	7 (36.8)	1 (7.1)	0.098
PHQ-9 ≧10, %	7 (21.2)	2 (10.5)	5 (35.7)	0.106
Forced crying or laughing, (%)	6 (18.2)	4 (21.1)	2 (14.3)	1.000
Pressure ulcer, (%)	2 (6.1)	1 (5.3)	1 (7.1)	1.000

*PHQ-9* Patient Health Questionnaire-9

^a^Comparison between patients and their families was performed using Fisher's exact test for categorical data and Mann–Whitney U test for continuous data. Bold *P* value < 0.05 between male and female

**Table 3:** Relationship between non-motor symptoms and PHQ-9 or ALSFRS-R scores

	Symptoms		*P* value^a^
	+	−	
Fatigue,* n*	27	6	
ALSFRS-R	33.0	22.5	0.575
PHQ-9	4.0	1.0	0.105
Pain,* n*	20	13	
ALSFRS-R	32.0	32.0	0.658
PHQ-9	5.0	2.0	**0.014**
Defecation disorder,* n*	19	14	
ALSFRS-R	20.0	34.0	0.083
PHQ-9	4.0	4.0	0.105
Sleep disorder,* n*	16	17	
ALSFRS-R	33.5	27.0	0.387
PHQ-9	5.0	4.0	0.054
Sialorrhea,* n*	16	17	
ALSFRS-R	16.0	34.0	**0.031**
PHQ-9	4.0	4.0	0.502
Sexual problems,* n*	8	25	
ALSFRS-R	33.5	29.5	0.207
PHQ-9	4.0	4.0	0.688
PHQ-9 ≧10,* n*	7	26	
ALSFRS-R	33.5	29.5	0.843
Decrease in QoL due to one or more non-motor symptoms	25	8	
ALSFRS-R	32	29	0.817
PHQ-9	4.0	1.5	**0.016**

*ALSFRS-R* Amyotrophic Lateral Sclerosis Functional Rating Scale-Revised, *QoL* quality of life, *PHQ-9* Patient Health Questionnaire-9

^a^Comparison between patients and their families was performed using Fisher's exact test for categorical data and Mann–Whitney U test for continuous data. Bold *P* value < 0.05 between with symptom and without symptom group

**Table 4:** Details of each non-motor symptoms among patients with ALS

Patients who reported pain, (*n* = 20)		Median ALSFRS-R
Muscle cramps, %	8 (40.0)	35.5
Hypertonia, %	7 (35.0)	27.0
Joint contracture, %	5 (25.0)	27.0
Pressure due to immobility, %	8 (40.0)	9.5
Cause unknown, %	6 (30.0)	34.5

Patients who reported sleep disorders, (*n* = 16)		Median ALSFRS-R
Muscle cramps, %	5 (31.3)	42.0
Anxiety, %	4 (25.0)	38.0
Pain (Excludes muscle cramps), %	4 (25.0)	6.5
Dyspnea (including sleep apnea), %	4 (25.0)	36.5
Cause unknown, %	9 (56.3)	31.0

Patients who reported defecation disorders, (*n* = 20)		Gastrostomy		Median ALSFRS-R
		+	−	
Constipation, %	13 (68.4)	5	8	32.0
Diarrhea, %	3 (15.8)	2	1	N/A
No urge to defecate, %	2 (10.5)	0	2	N/A
Lack of abdominal pressure, %	11 (57.9)	8	3	9.5
Feeling of residual stool, %	6 (31.6)	3	3	25.5
Fecal incontinence, %	3 (15.8)	2	1	N/A

Patients who reported sexual problems (*n* = 8)	Number of patients with sexual problems/total number of patients	Percentage of patients
Aged < 40 years	2/3	66.7
Aged 40–49 years	0/1	N/A
Aged 50–59 years	2/7	28.6
Aged ≥ 60 years	4/22	18.2

Multiple answers were allowed for questions about the causes of non-motor symptoms

*ALSFRS-R* Amyotrophic Lateral Sclerosis Functional Rating Scale-Revised, *N/A* Not Available

## Discussion

Few studies have comprehensively investigated non-motor symptoms of ALS. This cross-sectional study investigated the frequency of non-motor symptoms of ALS. Most ALS patients have some form of non-motor symptoms, many of which decrease their QoL. In this study, the frequency of non-motor symptoms other than sialorrhea was not affected based on the ALSFRS-R, the severity focused on motor symptoms. We highlight this as important. Therefore, non-motor symptoms may lower QoL even if the motor symptoms of ALS are mild, and should always be noted.

### Fatigue and pain

Fatigue is a common non-motor symptom in ALS, and previous reports have shown that approximately 50% − 80% of ALS patients have fatigue [10, 11]. Pain is also a common non-motor symptom of ALS, with a frequency reported to be 56% − 70% [12]. In this study, approximately 80% of the patients had fatigue and 60% had pain, similar to previous reports. On the other hand, no difference in the prevalence of fatigue by sex has been reported [10], but it should be noted that it was more common in females in this study. Some studies have reported that females experience more fatigue even with diseases such as myasthenia gravis and depression [13, 14]. Although a hormonal origin has been suggested, no significant conclusions have been reported. Furthermore, the PHQ-9 score was higher in the pain group in this study, consistent with previous reports [15] that pain is associated with depression. The median PHQ-9 was 3.0 higher in the symptomatic group, although there was no significant difference in fatigue. Furthermore, approximately 70% of the patients with ALS answered that their symptoms decreased their QoL in the group with fatigue and pain. In particular, we can treat various issues related to pain. ALS pain is multifactorial, and a variety of treatments have been proposed, including nonsteroidal anti-inflammatory drugs, neuropathic pain drugs, opioids, and physiotherapy strategies. In this study, the causes of pain were various, and the main factors were muscle cramps and compression due to immobility. This should also consider the effects of motor symptoms. Analgesics, such as opioids, are often adequately used in late-stage ALS patients, but as mentioned above, pain may appear early in ALS. Therefore, considering the decrease in QoL associated with pain and its effect on mental health, we need to actively respond with appropriate treatment for pain.

### Sleep disorder

In previous studies, 50%–63% of patients with ALS reported poor sleep quality [16]. In our study, the frequency of sleep disorders was the same as in previous reports, and more than 60% of ALS patients with sleep disorders answered that their QoL decreased due to sleep disorders. Sleep disorders in ALS are caused by a variety of factors, some of which have been reported to be associated with REM sleep behavioral disorders [17]. Additionally, respiratory sounds, such as TIV and dyspnea, may be one of the causes. In our study, however, there was no association between ALSFRS-R score and sleep disorders, and even low scores of ALSFRS-R were not associated with sleep disorders. On the other hand, none of the patients mentioned RBD as a detail of their sleep disorders in our study. The causes of sleep disorders are most often unknown, and ALS patients with sleep disorders were found to be vaguely unable to sleep. The PHQ-9 scores tended to be higher in patients with sleep disorders, although not significantly different, and might be associated with depression. There are few reports on the effects of sleep disorders on the QoL of patients with ALS. Long-term clinical studies are essential to establish the importance of sleep disorders in patients with ALS.

### Defecation disorder

No previous reports have focused solely on defecation dysfunction associated with ALS. However, there are reports of gastrointestinal disorders including nausea [18]. In our study, 57.6% of the patients with ALS had defecation disorders. In addition to constipation, defecation disorders included diarrhea, loose stools, hard stools, fecal incontinence, and poor abdominal pressure, and the frequency was almost the same as that previously reported for other gastrointestinal issues. Defecation disorders in ALS are caused by inadequate fiber intake and dehydration due to dysphagia and lack of physical activity due to motor symptoms. It has also been suggested that the autonomic nervous system is affected in ALS patients [19, 20]. In this study, the median ALSFRS-R score was 14 points lower in the group with defecation disorders, and it is possible that they were affected by motor symptom. The median ALSFRS-R for constipation, the most common symptom, was not low, although the motor symptom, inability to apply abdominal pressure, was also mixed. On the other hand, the fact that there was no significant difference may indicate some pathology of ALS affect the autonomic nervous system in those patients who have had defecation disorders.

### Sialorrhea

Sialorrhea was found in approximately 50% of our patients, which was as frequent as in previous studies [21]. Sialorrhea is caused primarily by a decreased ability to swallow secretions due to tongue spasticity, failure of orofacial and palatolingual muscle control, facial weakness, and inability to maintain oral and buccal capacity [22]. The effects of ALS on the autonomic nervous system have also been suggested [20], but most are attributed to motor symptoms. In this study, patients with sialorrhea had low ALSFRS-R scores and may have had complications associated with motor symptoms.

### Sexual problems

To our knowledge, the proportion of people with sexual problems has been rarely reported. In this study, more than 90% of patients with ALS answered that they had no place to discuss this important issue. The question also included not only sexual function disturbances, but sexuality-related problems such as sexual activity. The high proportion of young patients under the age of 40 who answered they have these problems may require attention to sexuality-related problems. Previous study described that over 75% of clinicians reported that they were not familiar with any strategies or interventions to help patients with sexuality-related problems [23]. In our study, there was no difference in whether there was a place to consult for sexual problems depending on whether the patient visited the ALS multidisciplinary clinic. Currently, the ALS multidisciplinary clinic should be the recipient of this problem; however, we assume that it is not. We recommend identifying issues that may exist when visiting the ALS clinic and taking action such as counseling [24].

### Depression

Other studies have reported a weak association between physical disabilities in ALS and depression [25]. However, on the other hand, some studies have shown an association between the degree of physical disability of ALS and depression [26]. The relationship between low ALSFRS-R scores and high PHQ-9 scores was weak in our study. Additionally, The PHQ-9 score was high in the group with pain and tended to be higher in the group with sleep disorders. Therefore, our study suggest that non-motor symptoms affect PHQ-9 rather than motor symptoms.

### QoL

Seventy percent of ALS patients in the group with fatigue and pain reported that their symptoms decreased QoL, and in the group with sleep and defecation disorders, more than 50%. The relationship between low ALSFRS-R scores and decrease in QoL was weak in our study. Furthermore, ALS patients who answered that There was a decrease in QoL due to non-motor symptoms had a high PHQ-9 score. These findings are consistent with previous reports that non-motor symptoms rather than severe physical disability may lower QoL [4].

## Limitations

The first limitation is that our study was a cross-sectional study based on a questionnaire survey, with a small number of respondents. Furthermore, since we could not identify ALS patients among the participants in the webinar, it was difficult to determine the response rate. Multicenter collaboration is needed to increase the number of participants with ALS, a rare disease, and long-term prospective studies are required to increase credibility of the findings. Second, the evaluation of QoL was based on the answers from our own questionnaire. While preexisting measures of QoL such as the sickness impact profile / ALS-19 and ALS Assessment Questionnaire-40 or 5 could have been used, we needed to reduce the number of questions in consideration of the burden on ALS patients due to the time to complete questionnaires. In addition, our study did not investigate the duration of illness and cognitive function, a typical non-motor symptom of ALS. Since this was a questionnaire-style survey on the web, it was difficult to accurately evaluate the duration of illness, and cognitive function was not evaluated in terms of the accuracy of the questionnaire responses. On the other hand, there are no comprehensive studies on the non-motor symptoms of ALS, other than review articles that collect multiple studies of limited non-motor symptoms. We highlight that this is the strength of our study.

## Conclusion

We found that most patients with ALS had some non-motor symptoms, especially the high frequency of fatigue and pain. They may appear independently of the progression of motor symptoms of ALS and decrease the QoL. However, it should be noted that some causes of non-motor symptoms may be caused by motor symptoms. There are also infrequently studied non-motor symptoms, such as defecation disorders and sexual problems, and many ALS patients, in particular, have no place to discuss sexual problems that are difficult to identify. It is necessary to identify and address this problem based on future long-term investigations.

## Data Availability

The data that support the findings of this study are available on request from the corresponding author, TH. The data are not publicly available due to containing information that could compromise the privacy of research participants.

## Abbreviations

ALS: Amyotrophic lateral sclerosis
QoL: Quality of life
ALSFRS-R: ALS functional rating scale-revised
PHQ-9: Patient health questionnaire-9
TIV: Tracheostomy and invasive ventilator
